# Association of plasma gelsolin with frailty phenotype and mortality among octogenarian community-dwelling men: a cohort study

**DOI:** 10.1007/s40520-022-02083-2

**Published:** 2022-02-15

**Authors:** Timo E. Strandberg, Susan L. Levinson, Mark J. DiNubile, Satu Jyväkorpi, Mika Kivimäki

**Affiliations:** 1 Helsinki University Hospital, HUS, University of Helsinki, PO Box 340, FI-00029 Helsinki, Finland; 2 Center for Life Course Health Research, University of Oulu, Oulu, Finland; 3 BioAegis Therapeutics Inc, North Brunswick, NJ USA; 4 Clinicum, Faculty of Medicine, PO Box 20, FI-00014 Helsinki, Finland

**Keywords:** Biomarker, Prefrailty, Sarcopenia

## Abstract

**Background:**

Biomarkers are needed for frailty, a common phenotype often associated with muscle loss in older people. Plasma gelsolin (pGSN) is a protein largely synthesized and secreted by skeletal muscle.

**Aims:**

To investigate whether pGSN could be a biomarker of the frailty phenotype and predict mortality.

**Methods:**

A homogenous cohort of males (born 1919–1934, baseline *n* = 3490) has been followed since the 1960s. In 2010/11, frailty phenotypes by modified Fried criteria were assessed. pGSN was measured in a convenience subset (*n* = 469, mean age 83) and re-measured in survivors (*n* = 127) in 2017. Mortality through December 31, 2018 was retrieved from national registers. Regression models were used for analyses.

**Results:**

Of 469 males, 152 (32.4%) were robust, 284 (60.6%) prefrail, and 33 (7.0%) frail in 2010/11. There was a graded (*p* = 0.018) association between pGSN (mean 58.1 ug/mL, SD 9.3) and frailty. After multivariable adjustment, higher pGSN levels were associated with lower odds of having contemporaneous phenotypic prefrailty (OR per 1 SD 0.73, 95% CI 0.58–0.92) and frailty (OR per 1 SD 0.70, 95% CI 0.44–1.11). By 2018, 179 males (38.2%) had died, and higher baseline pGSN predicted a lower 7-year mortality rate (HR per 1 SD 0.85, 95% CI 0.72–1.00). pGSN concentrations in 2010/11 and 2017 were correlated (*n* = 127, *r* = 0.34, *p* < 0.001).

**Discussion:**

Higher baseline pGSN concentrations were associated with a persistently robust phenotype and lower mortality rate over 7 years in a cohort of octogenarian males with high socioeconomic status and may be a promising laboratory biomarker for the development of a frailty phenotype.

## Introduction

The calcium-dependent role of gelsolin in the regulation of the actin cytoskeleton was first described by Yin and Stossel in 1979 [1, 2]. However, numerous other functions of gelsolin in intracellular and extracellular compartments have also been discovered [2–4]. Gelsolin belongs to the gelsolin superfamily and 3 isoforms have been identified: cytoplasmic gelsolin, plasma gelsolin (pGSN), and gelsolin-3 [4]. pGSN is primarily involved in the rapid removal of actin filaments and other debris released from dead cells into the circulation. In addition, it binds and sequesters various proinflammatory and bioactive substances and may therefore be involved in physiological and pathological functions like wound healing, immune and inflammatory reactions, angiogenesis and cancer progression [2–4]. Consequently, gelsolin is a potential candidate for both diagnostic and therapeutic purposes [2]. Lower pGSN values have been observed in diverse infectious diseases, major trauma, rheumatic diseases, neurological conditions, and cancer [4–6]. Replenishment of depleted pGSN in animal models of inflammation can improve survival and diminish tissue injury [7–11].

Because skeletal muscle is a major source of gelsolin circulating in serum [12], we hypothesized that reduced pGSN could act as a biomarker for the frailty phenotype often associated with loss of muscle mass, whereas higher pGSN might be related to robustness and lower mortality. We tested these hypotheses in a cohort of octogenarian men participating in a follow-up study (the Helsinki Businessmen Study [HBS]).

## Methods

### Participants

The HBS cohort has been described in detail previously [13, 14], and the follow-up has been approved by the ethical committee of the Department of Medicine of our University Hospital.

In brief, 3490 men, mostly business executives born between 1919 and 1934, participated in volunteer health check-ups during the 1960s and early 1970s. The cohort has been followed-up using national registries, regular postal questionnaire surveys with good response rates, and clinical examinations in random subcohorts. The present analysis originated from a convenience sample of 525 home-dwelling men who were investigated with questionnaires (including the RAND-36/SF-36 health-related quality of life instrument and history of physician-diagnosed chronic diseases) and clinical examinations including laboratory tests in 2010/11, with subsequent 7-year mortality follow-up. Of the 525 men, we had plasma gelsolin available for 505 men, and the frailty phenotype could be assessed in 469 men. The 469 men (89% of 525) form the analytical group for the present mortality and frailty analyses.

Multimorbidity was defined as the presence of at least 2 out of 8 chronic diseases [15]: coronary artery disease (chronic or history of myocardial infarction), stroke, peripheral artery disease, heart failure, chronic pulmonary disease (including asthma and chronic obstructive pulmonary disease), diabetes (type not defined, but majority likely type 2), any cancer and musculoskeletal diseases (including rheumatic diseases and osteoarthritis).

### Clinical and laboratory investigations

After the mailed survey in 2010/11 we clinically studied 525 survivors willing to visit the clinic, completing the investigation with a blood venous sample for laboratory analyses. Because the time between postal questionnaire and clinical examination varied, contemporary lifestyle, mental, and functional data were updated through an interview by the study nurse.

The clinical investigations included measured height, weight, waist circumference, blood pressure, pulse rate, tests for physical function (peak expiratory flow [PEF], walk speed (m/s, usual pace), handgrip (a handheld dynamometer), and Mini-Mental State Examination (MMSE). Weight-adjusted waist index, reflecting fat and muscle mass, was calculated as previously described [16]. Routine clinical instruments were used, and a limited set of laboratory variables were assayed from plasma after a 12-h fast, including lipids, glucose, insulin, creatinine, high-sensitivity C-reactive protein (hs CRP), alanine aminotransferase (ALT), homocysteine, and prealbumin. The analyses were performed with standard methods at the clinical laboratory of the University Hospital. pGSN from frozen samples was measured by BioAegis Therapeutics, Inc (North Brunswick, NJ) using a proprietary enzyme-link immunosorbent assay (ELISA). Median pGSN concentration in control plasma samples from healthy adults was 56.8 μg/mL (interquartile range [IQR], 52.6–65.4 μg/mL. Laboratory personnel who performed the assay were blinded to clinical outcomes [6].

In 2017, the questionnaire, clinical and laboratory examinations including pGSN measurements were repeated for 127 men of the cohort. As in 2010/11, this was a convenience sample of HBS survivors willing to visit the clinic. Accordingly, they represent a group of well or relatively well-functioning survivors.

### Frailty assessment

Robust, prefrailty and frailty phenotype were defined in 469 men according to the 5 criteria described in the Cardiovascular Health Study (CHS) by Fried et al. [17], with the following modifications:

1. Shrinking: Weight loss > 5% between 2007 (closest data before 2010/11) and 2010/11, or current BMI < 22.0 kg/m^2^.
2. Weakness: Hand-grip strength (cut point 27 kg) [18].
3. Exhaustion (reported low energy most or all of the time during the preceding 4 weeks; one of the questions of the Vitality domain of RAND-36).
4. Low activity (based on the question: “Do you exercise regularly weekly?”; the response “no” was taken to denote low physical activity.
5. Slowness (walking speed < 0.8 m/s) [19].

Responders with 3–5, 1–2, and zero criteria were classified as frail, prefrail, and robust, respectively.

In the 2017 examinations, frailty status was defined as above, except that weight loss was calculated from weight measured in 2010/11.

### Mortality

Participants were linked to electronic health records of the Digital and Population Data Services Agency. This agency keeps a registry of all citizens in our country and thus determination of vital status was reliable with no loss to follow-up. We retrieved the total mortality of the study cohort through December 31, 2018.

### Statistical analyses

Statistical analyses were performed with NCSS 2020 software (NCSS, Kaysville, Utah). Continuous variables are shown as means with standard deviation (SD), standard error (SE), or medians with interquartile ranges (IQRs). Spearman rank correlation and analysis of covariance (ANCOVA) were used to compare groups. Logistic regression was used to examine associations of gelsolin and other baseline covariates with frailty and prefrailty. Covariates were clinically meaningful variables which in univariate analyses were significantly related to frailty phenotype (*p* < 0.05). pGSN was studied both as a continuous variable and as divided into thirds with the bottom tertile as the reference. The results are presented as odds ratios (OR) with their 95% confidence intervals (CI).

After confirming that the proportional hazards assumption was met, predictors of 7-year mortality were examined using Cox proportional hazards models. Results are presented as hazard ratios (HR) with their 95% CIs. To assess the independent role of gelsolin, effect estimates were adjusted for age and frailty phenotype in 2010/11. Receiver operating characteristic (ROC) curve was constructed for pGSN and frailty. In all analyses, p-values < 0.05 (two-sided) were considered to indicate statistical significance.

## Results

Of the analytical sample (*n* = 469) in 2010/2011 (mean/median age at entry 83.5/82 years, IQR 80–85), 152 (32%), 284 (61%) and 33 (7%) were assessed to be robust, prefrail and frail, respectively. Baseline characteristics according to frailty groups are shown in Table 1. Clinical characteristics related to the frailty phenotype (walk speed, grip strength, PEF) were lower, while the reported number of falls during the past year was higher among frail than robust men. During the 7-year follow-up, 179 (38%) men died and the frailty phenotype was significantly and in a graded fashion (*p* < 0.001) associated with mortality risk (Table 1). While BMI and waist circumferences were similar in the groups, weight-adjusted waist circumference was significantly different (*p* = 0.023) between the groups. Of the routine laboratory variables, only plasma homocysteine levels differentiated robust men (lower homocysteine) compared with prefrail and frail men (p = 0.016 between groups). hs CRP concentration and lipid values were not significantly different between frailty groups.

**Table 1:** Age-adjusted characteristics (mean [SE]) according to frailty phenotype at baseline in 2010/11

Variable^a^	All, *n* = 469	Robust *n* = 152 (32.4%)	Prefrail *n* = 284 (60.6%)	Frail, *n* = 33 (7.0%)	*p*-value for linear trend between frailty groups
Age, yr	83.5 (0.2)	81.5 (0.2)	83.1 (0.2)	85.8 (0.6)	< 0.001
Multimorbidity ^b^, *n* (%)	168 (35.8)	49 (32.2)	104 (36.8)	15 (45.5)	0.072
BMI, kg/m^2^	25.0 (0.2)	24.8 (0.2)	25.1 (0.2)	25.0 (0.4)	0.60
Waist circumference, cm	96.6 (2.6)	96.3 (2.9)	97.8 (2.5)	95.7 (5.3)	0.20
Weight-adjusted waist index	10.9 (0.04)	10.8 (0.05)	11.0 (0.04)	10.9 (0.09)	0.023
Smokers, *n* = 18, %	3.8	3.3	2.8	15.2	0.002
Plasma gelsolin, ug/mL	58.1 (0.4)	59.9 (0.8)	57.4 (0.6)	56.2 (1.6)	0.018
Plasma lipids, mmol/L					
Cholesterol	4.8 (0.07)	4.9 (0.08)	4.7 (0.06)	4.7 (0.1)	0.37
HDL cholesterol	1.6 (0,03)	1.6 (0.03)	1.5 (0.03)	1.6 (0.06)	0.34
LDL cholesterol	2.7 (0.05)	2.8 (0.06)	2.6 (0.05)	2.6 (0.1)	0.37
Triglycerides	1.26 (0.04)	1.29 (0.05)	1.28 (0.04)	1.20 (0.09)	0.74
Plasma glucose, mmol/L	5.8 (0.09)	5.9 (0.1)	5.8 (0.09)	5.7 (0.2)	0.89
Plasma insulin, mU/L	15.2 (0.9)	12.5 (1.1)	15.0 (0.9)	18.0 (2.0)	0.11
Plasma urate, umol/L	383.4 (6.1)	379.2 (6.6)	387.1 (5.6)	396.0 (12.1)	0.57
Prealbumin, mg/L	249.6 (3.0)	256.2 (3.0)	253.9 (3.0)	238.7 (6.0)	0.16
hs CRP, mg/L	2.4 (0.4)	1.9 (0.5)	3.2 (0.4)	2.3 (0.8)	0.37 (log)
Creatinine, umol/L	96.1 (2.0)	94.6 (2.3)	99.7 (2.0)	93.9 (4.2)	0.22
ALT, U/L	18.3 (0.5)	19.8 (0.6)	18.3 (0.5)	16.9 (1.1)	0.11
Homocysteine, umol/L	15.7 (0.4)	14.9 (0.4)	16.3 (0.3)	16.7 (0.7)	0.016
PEF	429.5 (6.0)	464.2 (6.5)	430.9 (5.6)	393.4 (12.7)	< 0.001
MMSE, points	28.0 (0.1)	28.5 (0.2)	28.2 (0.1)	27.5 (0.3)	0 .06
Walk speed, m/s	0.81 (0.01)	0.97 (0.01)	0.78 (0.01)	0.67 (0.02)	< 0.001
Grip strength, kg	34.5 (0.7)	39.7 (0.7)	36.4 (0.6)	27.5 (1.3)	< 0.001
7-year mortality, *n* (%)	179 (38.2)	31 (20.4)	125 (44.0)	23 (69.7)	< 0.001
No reported falls during past year *n* (%)	319 (68.1)	121 (79.6)	182 (64.1)	16 (48.5)	< 0.001

Log-transformed values where used where indicated

*ALT* alanine aminotransferase, *BMI* body mass index, *hsCRP* high-sensitivity C-reactive protein, *MMSE* Mini-mental state examination, *PEF* peak expiratory flow

^a^Age-adjusted, continuous variables are means (SE)

^b^At least 2 out of 8 of important diseases as described in “Methods”

Mean and median pGSN levels in the analytic sample were 58.1 ug/mL (SE 0.4) 57.9 ug/mL (IQR 52.2 to 64.0), respectfully and followed a Gaussian distribution. pGSN levels did not correlate with age (Spearman coefficient = 0.004, *p* = 0.93), but there was a graded and statistically significant (*p* = 0.018) association between frailty status and gelsolin level, with robust men having the highest values (Table 1). After adjustment for age, weight-adjusted waist circumference, smoking, homocysteine, and PEF, 1 SD higher pGSN level was associated with a 27% lower odds of prefrailty phenotype (OR 0.73; 95% CI 0.58–0.92), whereas the 30% lower odds of frailty phenotype was not statistically significant (OR 0.70, 95% CI 0.44–1.11) (Table 2).

**Table 2:** Odds ratios of prefrailty and frailty phenotype according to plasma gelsolin concentration (*N* = 469)

Adjustment	Odds ratio (95% CI) per 1 SD increase in plasma gelsolin (robust group as reference = 1.0)	
	Prefrail *n* = 284	Frail *n* = 33
Unadjusted	0.79 (0.65–0.96)	0.73 (0.50–1.07)
Age	0.74 (0.63–0.94)	0.65 (0.43–0.97)
Age, weight-adjusted waist circumference and homocysteine	0.74 (0.59–0.91)	0.63 (0.41–0.96)
Age, weight-adjusted waist circumference, homocysteine, smoking, and peak expiratory flow	0.73 (0.58–0.92)	0.70 (0.44–1.11)

*95% CI* 95% confidence interval, *SD* standard deviation

We also compared the odds of frailty according to tertiles of pGSN concentration (Table 3). In a model adjusted for age, weight-adjusted waist circumference, smoking, homocysteine, and PEF, using the bottom tertile as referent, the top tertile was associated with a 50% lower odds of prefrailty phenotype (OR 0.50, 95% CI 0.28–0.89). Point estimates for higher tertiles and frailty were well below unity, but not statistically significant (Table 3). In the ROC analysis, the area under the curve for pGSN level and frailty was 0.68 (95% CI 0.59–0.75).

**Table 3:** Odds ratios of prefrailty and frailty phenotype in octogenarian males according to plasma gelsolin tertiles (N = 469)

Gelsolin tertiles	*N* (cases)	Odds ratio (95% confidence interval)	
		Model 1 ^a^	Model 2 ^b^
		Outcome: Prefrailty	
Bottom	154 (103)	1.00 (reference)	1.00 (reference)
Intermediate	165 (96)	0.67 (0.42–1.08)	0.76 (0.43–1.32)
Top	150 (85)	0.68 (0.42–1.10)	0.50 (0.28–0.89)
		Outcome: Frailty	
Bottom	154 (12)	1.00 (reference)	1.00 (reference)
Intermediate	165 (13)	0.71 (0.34–1.50)	0.70 (0.24–1.99)
Top	150 (8)	0.24 (0.09–0.60)	0.38 (0.12–1.21)

^a^Adjusted for age

^b^Adjusted for age, weight-adjusted waist circumference, homocysteine, smoking, and peak expiratory flow

During the 7-year follow-up, 31 (20%), 125 (44%), and 23 (70%) men died in the robust, prefrail, and frail groups, respectively (*p* < 0.001). After adjustment for age, higher pGSN, both as a continuous (per SD) and as a categorical variable in tertiles, was associated with a significantly lower risk of mortality (Table 4). Higher pGSN concentration also tended to predict mortality independently of the frailty phenotype (HR per 1 SD 0.85, 95% CI 0.72–1.00).

**Table 4:** Hazards ratios of 7-year survival according to plasma gelsolin among octogenarian males (*N* = 469)

		Hazards ratio (95% confidence interval)	
		Outcome: Mortality	
Gelsolin, continuous per SD	*N* (dead)	Model 1^a^	Model 2^b^
	469 (179)	0.84 (0.72–0.99)	0.85 (0.72–1.00)
Gelsolin tertiles	*N* (dead)	Model 1^a^	Model 2^b^
Bottom	154 (69)	1.00 (reference)	1.00 (reference)
Intermediate	165 (59)	0.75 (0.52–1.08)	0.76 0.53–1.09)
Top	150 (51)	0.64 (0.43–0.93)	0.69 (0.47–1.01)

^a^Adjusted for age

^b^Adjusted for age and frailty phenotype in 2010/11

In the 2017 examination of 127 surviving men (mean age 89 years, SD 2.7), 35 (27%) were robust, 81 (64%) prefrail, and 11 (9%) frail, and the groups are compared in Table 5. pGSN concentrations in 2010/11 and 2017 were correlated (Spearman coefficient = 0.34, *p* < 0.001). Also in 2017 there was a graded association between mean age-adjusted gelsolin concentrations and frailty status but in this smaller group of survivors the association was not statistically significant (*p* = 0.33).

**Table 5:** Follow-up characteristics according to frailty phenotype in 2017

Variable^a^	Frailty phenotype in 2017				
	All, *n* = 127	Robust *n* = 35 (27.6%)	Prefrail *n* = 81 (63.8%)	Frail, *n* = 11 (8.7%)	*p*-value for linear trend between frailty groups
Age, year	88.6 (0.4)	88.0 (0.3)	88.2 (0.3)	89.7 (0.8)	0.17
Plasma gelsolin in 2010/11, ug/mL, *n* = 118	57.7 (1.2)	60.6 (1.5)	57.7 (1.0)	54.2 (2.7)	0.09
Plasma gelsolin in 2017, ug/mL	62.7 (1.4)	65.3 (1.9)	62.4 (1.3)	60.1 (3.2)	0.33
Frailty phenotype in 2010/11^b^, %					0.08 between groups
-robust, *n* = 52		28.9	67.3	3.9	
-prefrail, *n* = 48		18.8	64.6	16.7	
-frail, *n* = 2		0	50	50	

^a^Age-adjusted, continuous variables are mean (SE)

^b^Both 2010/11 and 2017 frailty status available in 102 men

## Discussion

In our octogenarian, home-dwelling male cohort, a higher pGSN level was associated with a robust phenotype independently of other clinical characteristics associated with frailty. Higher baseline pGSN level also predicted lower total mortality over 7 years of follow-up. Among a large set of laboratory variables, including hsCRP, pGSN was a strong independent biomarker for the frailty phenotype. This independency suggests that lower pGSN values are mainly due to lower muscle mass and reduced production rather than increased consumption by low-grade inflammation related to aging (“inflammaging” [20]). Studies examining gelsolin and mortality are relatively scarce and mainly restricted to clinical samples, such as patients with severe and usually acute conditions [2–5]. To the best of our knowledge, there are no previous studies on the association between gelsolin and frailty phenotype. Therefore, our results in community-dwelling older men should stimulate further studies to examine whether our findings in this socioeconomically and ethnically restricted cohort would be reproducible across different study populations and settings.

The robust association between low gelsolin and frailty is important because reliable laboratory biomarkers of phenotypic frailty for clinical use are currently lacking. Operational definition of frailty phenotype [17] based on five non-laboratory criteria had prognostic validation also in our male cohort. However, there is a constant search for laboratory biomarkers [21] which could be helpful in screening the condition and give insights into the pathogenesis of frailty phenotype.

Because pGSN largely originates from myocytes [12], our results suggest that gelsolin reflects frailty as a condition with reduced muscle mass [22]. We did not directly measure muscle mass in the present study. However, in-body measurements of body composition were performed for a random subcohort of 87 men in 2010/11, and among them gelsolin was significantly correlated with lean body mass (Spearman coefficient = 0.29, *p* = 0.006).

There are no established reference values for pGSN in an older population; in our ELISA analysis, the median concentration of healthy adults has been 56.8 ug/mL, falling in the range of the present older cohort (Table 1). The pGSN concentrations showed a Gaussian distribution and were higher than in acute pneumonia patients [6]. In a small series of surgical patients, plasma gelsolin concentration below 61 ug/mL was associated with increased mortality risk [5]. Considering the origin of pGSN from muscle and increasing prevalence of muscle loss with age, it would not be surprising if concentrations were lower in octogenarians. Those concentrations did not materially change irrespective of phenotype during our follow-up may be explained by attrition: only better-functioning survivors were alive and able to participate in clinical examinations at a mean age of 88 years. On the other hand, pGSN concentrations did not vary much with increasing age [23], and accordingly in our cohort, pGSN and age in 2010/2011 were not significantly correlated.

Limitations of our study include the inclusion of a community-dwelling all-male-all white cohort with high socioeconomic status—limiting generalizability—and the lack of details regarding comorbidities and hospitalizations. Further studies including women and non-white participants are warranted to examine the generalizability of our findings. We did not define frailty phenotype using the exact CHS criteria, but the validity of our definition was supported by its predictive validity for mortality and association with fall tendency. Gelsolin values in our cohort were higher than concentrations in severe diseases probably reflecting the relatively high functional status of the participants (only half with frailty phenotype were frail according to frailty index, unpublished observations). Area under the curve was significantly high considering that gelsolin was used as a single biomarker of a multi-factorial frailty phenotype. Future studies are needed to find a possible frailty biomarker score where gelsolin could be one important component. Finally, our analyses cannot distinguish cause and effect, i.e., exclude the possibility that high pGSN would actually protect from frailty. This directionality would be plausible considering the pleiotropic effects of gelsolin [2–4]. A physiological role for pGSN in normal aging would have potential therapeutic implications.

## Conclusions

Our results suggest that plasma gelsolin predicts long-term mortality among octogenarian males and is a promising biomarker for phenotypic frailty. These findings should be considered hypothesis-generating rather than definite as our study was based on a relatively small sample size and did not include replication in an independent population.

## Data Availability

From authors upon appropriate request.
